# Research on the Gravity Disturbance Compensation Terminal for High-Precision Position and Orientation System

**DOI:** 10.3390/s20174932

**Published:** 2020-08-31

**Authors:** Zhuangsheng Zhu, Hao Tan, Yue Jia, Qifei Xu

**Affiliations:** 1 Research Institute for Frontier Science, Beihang University, Beijing 100191, China; hhtan@buaa.edu.cn (H.T.); jiayue@buaa.edu.cn (Y.J.); leisin_xqf@163.com (Q.X.); 2 Key Laboratory of Fundamental Science for National Defense-Novel Inertial Instrument & Navigation System Technology, Beijing 100191, China

**Keywords:** POS, gravity compensation, INS/GNSS, vertical deflection, gravity measurement

## Abstract

The Position and Orientation System (POS) is the core device of high-resolution aerial remote sensing systems, which can obtain the real-time object position and collect target attitude information. The goal of exceeding 0.015°/0.003° of its real-time heading/attitude measurement accuracy is unlikely to be achieved without gravity disturbance compensation. In this paper, a high-precision gravity data architecture for gravity disturbance compensation technology is proposed, and a gravity database with accuracy better than 1 mGal is constructed in the test area. Based on the “Block-Time Variation” Markov Model (B-TV-MM), a gravity disturbance compensation device is developed. The gravity disturbance compensation technology is applied to POS products for the first time, and is applied in the field of aerial remote sensing. Flight test results show that the heading accuracy and attitude accuracy of POS products are improved by at least 6% and 16%, respectively. The device can be used for the gravity disturbance compensation of various inertial technology products.

## 1. Introduction

The Position and Orientation System (POS) is the core equipment of high-resolution aerial ground observation systems. It can measure the motion parameters of imaging load (such as Synthetic Aperture Radar, optical camera) accurately and autonomously, and provide high-precision information of time and space for aerial remote sensing imaging load in real-time [1,2,3].

The main architecture of the POS product is the Global Navigation Satellite System (GNSS) and Inertial Navigation System (INS) integrated system [4,5]. Its real-time output position and speed precision mainly depend on GNSS, and the real-time output attitude precision mainly depends on INS [6,7,8]. INS error mainly includes sensor error and model error. Sensor error mainly depends on sensor accuracy. Model error mainly includes sensor calibration model error and gravity model error [9,10]. Sensor calibration model technology [11,12] has essentially become mature, while the gravity model still adopts the most widely used normal gravity model, and particularly with the continuous improvement of the accuracy of the inertial sensor, the error of gravity model has become the main error source of high-precision INS [13].

**Figure 1:**
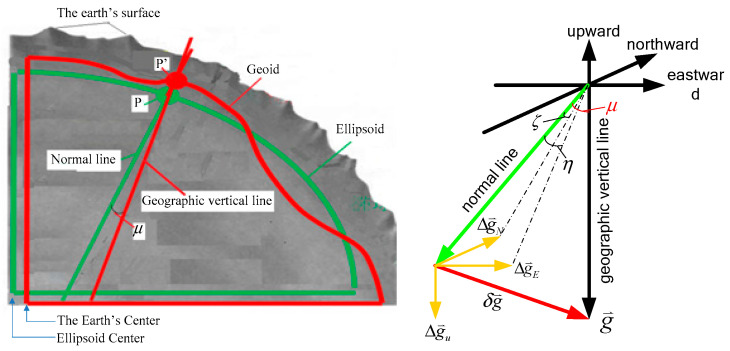
Gravity disturbance error (Reproduced From Figures 2 and 3 at Zhu, Z.S.; Zhao, B.; Guo, Y.Y. Research on Gravity Vertical Deflection on Attitude of Position and Orientation System and Compensation Method. Aerosp. Sci. Technol. 2019, 85, 495–504. Copyright © 2019 Elsevier Masson SAS. All rights reserved.).

The normal gravity model is based on a rotating ellipsoid with similar geoids. Different countries or regions choose different rotating ellipsoids. Helmert normal gravity formula is used in China:

$$\gamma =9.780300\left(1+0.005302\sin^{2}\varphi -0.000007\sin^{2}2\varphi \right)$$

where φ is the latitude. As shown in Figure 1, the normal gravity direction of any point of the earth follows a normal direction, while the actual gravity direction follows the geographic vertical line. There is a direction deviation μ between the normal gravity and the actual gravity. In a geographic coordinate system, the relationship between the two is as shown in Formula (1)

(1) $$\vec{g}=\vec{\gamma }+\delta \vec{g}=\left[\begin{array}{c} 0\\ 0\\ \gamma \end{array}\right]+\left[\begin{array}{c} \eta \gamma \\ \xi \gamma \\ \Delta g \end{array}\right]$$

In the formula, $\vec{g}$ is the actual gravity, $\vec{\gamma }$ is the normal gravity, $\delta \vec{g}$ is the gravity disturbance, ξ and η represent the deviation angles of the actual gravity and the normal gravity in the north and east directions respectively. In addition, Δg is the vertical component of the gravity disturbance $\delta \vec{g}$, and ηγ is the east component of the gravity disturbance $\delta \vec{g}$, and ξγ is the north component of the gravity disturbance $\delta \vec{g}$, and γ is the vertical component of the normal gravity $\vec{\gamma }$.

It can be seen from Formula (1) that the effect of gravity disturbance on the output of inertial measurement is mainly highlighted in the horizontal component, which mainly affects the attitude measurement accuracy of POS products. As shown in Table 1, the gravity disturbance of 25 mGal will result in a Pitch/Roll angle error of about 0.003° [9,14]. The variation range of ξ is about ~200″ in north-south and of η is about ~160″ in east-west [15]. Gravity disturbance has become the bottleneck of high-precision inertial measurement, and it is also the key technology with an urgent need to be developed for long endurance and high precision in INS [16,17].

The real-time acquisition of the gravity disturbance error is the key to the gravity disturbance compensation technology in real-time. There are two main real-time methods of access: the first is the direct access to the gravity sensor; the second is the model estimation method [9,14,18]. Gravity sensors [19,20] mainly include gravimeter and gravity gradiometer. The gravimeter can only obtain the vertical component of gravity disturbance in real-time, although the gravity gradiometer [21,22,23] can obtain the vertical component, the east component and the north component of the gravity disturbance at the same time. At present, it is only carried out on the satellite and fails to be used in the aviation field [24,25]. Therefore, in order to acquire the real-time gravity disturbance value in the aviation field, the model estimation method is currently put into wide usage. Since the current inertial products (POS or high-precision INS) have mature hardware and the research on gravity disturbance compensation technology is still in its infancy, the development of the gravity disturbance compensation device is carried out with the main purpose to make the gravity disturbance compensation technology independent of the inertial products, achieving improvement in the position and attitude measurement accuracy of the original inertial products without any change for the inherent structure of original products, and promising normal operation and the inherent accuracy of original products when faced with device failure.

The rest of this paper is organized as follows. In Section 2, the application-oriented high-precision gravity database architecture and precision evaluation method are introduced. Section 3 introduces the compensation method of gravity disturbance. Subsequently, the performance of the proposed method is verified through an actual test in Section 4. Subsequently, in Section 5, the gravity disturbance compensation device is detailed, and Section 6 includes conclusions and future work prospects.

## 2. High-Precision Gravity Database

A precise mastery of knowledge about the Earth’s gravity field structure with high resolution is essential for a range of disciplines, including mineral exploration and geophysics [26], climate and sea-level change research [27], and surveying and engineering [28]. While there is a strong scientific interest in model Earth’s gravity field with ever-increasing details, the resolution of the current gravity models is still insufficient for the application of the local gravity field, such as INS and water conservancy engineering. Therefore, in order to build a high-precision gravity database for the application of inertial technology, the existing gravity data must be reprocessed, so the Global Gravity Model plus (GGMplus) database with the highest precision at this stage [15] is selected as the basic database.

### 2.1. GGMplus

The GGMplus data provides an unprecedented ultrahigh-resolution view of Earth’s gravity field over all continents, coastal zones, and numerous islands within ±60° latitude. This is achieved through augmentation of new satellite and terrestrial gravity with topography data and the use of massive parallel computation techniques, delivering local detail at 7.2 arcsec (~220 m in north-south direction) spatial resolution. As such, it is the first of its kind to model gravity at ultrafine scales yet with near-global coverage. The new picture of Earth’s gravity encompasses a suite of gridded estimates of gravity accelerations, radial and horizontal field components, and quasi-geoid heights at over 3 billion points covering 80% of Earth’s landmass. The GGMplus is a combined solution based on the three key constituents GOCE (Gravity field and Ocean Circulation Explorer)/GRACE (Gravity Recovery and Climate Experiment) satellite gravity (providing the spatial scales of ~10,000 km down to ~100 km), EGM2008 (~100 km to ~10 km), and topographic gravity, i.e., the gravitational effect implied by a high-pass filtered terrain model (scales of ~10 km to ~250 m).

### 2.2. High-Precision Gravity Database for Inertial Measurement

It can be seen from Table 2 that the real-time attitude measurement accuracy of POS/AV610 products is 0.005° at present. Compared with the attitude measurement error caused by gravity disturbance error in Table 1, it can be seen that in order to realize gravity disturbance compensation, the accuracy of gravity disturbance compensation error should be less than 5 mGal. Considering that the error of GGMplus basic database in Asia is ~20 mGal, which fails to meet the application requirements of inertial technology, it is necessary to improve its accuracy.

The structure of GGMplus gravity database is a grid. Its abscissa is longitude and ordinate is latitude. Each grid point represents the known gravity point on the earth, and the points inside the grid are all unknown points. In addition, in order to meet the needs of inertial measurement technology and improve the accuracy of local gravity model data, it is necessary to upgrade the GGMplus model data database combined with the ground gravity observation data or gravity terrain model data in the test area. Therefore, the high-precision gravity database for inertial measurement technology mainly consists of the following data: The first layer is the basic database, and the data come from instrument measurement, mainly including GGMplus model data, ground observation data or terrain model data of the test area. The second layer is the average gravity database, which is derived from the smooth data of grid points. It mainly includes the interpolation data based on the basic database and the gravity data obtained by the direct difference method. The above-mentioned two-tier database format is in grid point form, which can not meet the requirements of inertial measurement technology. Therefore, we must build a function database, that is, the third layer database, in the form of a function expression. Therefore, the structure of the high-precision gravity database for inertial measurement technology is “Layered-Grid-Function” architecture.

### 2.3. Accuracy Evaluation of Gravity Database

(1) Selection of datum points for database accuracy evaluation

It can be seen from the above description that in order to generate a high-precision gravity database for inertial technology application, interpolation processing must be carried out on the basic database to improve the resolution of the database. Therefore, the gravity value obtained by interpolation must have interpolation model error. It is necessary to analyze the accuracy of the newly generated database after interpolation.

The evaluation datum points mainly come from two ways: one is from the gravity network reference point provided by the surveying and mapping department; the other is from the gravity disturbance data on the track line obtained by the aviation direct difference method.

(2) Determination of the precision index of gravity database

According to POS/AV610, the real-time accuracy index of attitude and heading angle is 0.005° and 0.03° respectively. In this paper, the error range of gravity database is −0.8 mGal~0.8 mGal, that is, attitude error caused by database error is better than 0.0001°.

(3) Evaluation rules and procedures

Since the data to be evaluated are discrete data in the form of grid points, it is difficult to correspond with the discrete grid points in the database, whether it is the datum points provided by the surveying and mapping department or the data points on the aircraft trajectory. Since the position accuracy of POS/AV610 is 4 m~6 m, the evaluation rules in this paper are as follows: taking the position of grid point as the center of the circle, the gravity disturbance value of any point in the circular area with radius of 6 m is equal to the gravity disturbance value at the grid point.

(4) Accuracy evaluation of database

In this paper, the Yanliang region, Changzhou region and the Hainan region are selected as the evaluation objects. In addition, the gravity data obtained by the direct aviation difference method are used as the datum point, and the evaluation interpolation points will be selected along the flight path.

**Figure 2:**
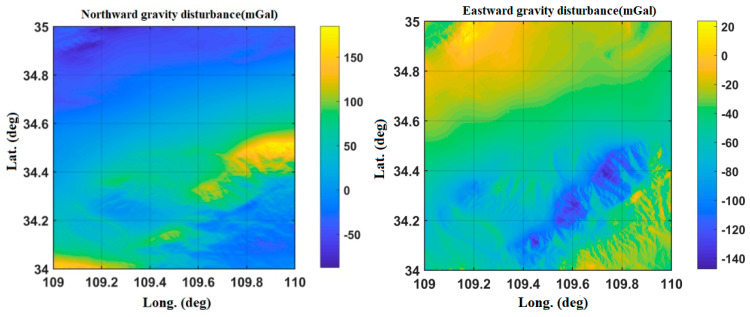
Variation of gravity disturbance in the Yanliang area.

As shown in Figure 2, the gravity disturbance database in Yanliang area is interpolated, with the highest value of about 160 mGal and the lowest value of −140 mGal.

**Figure 3:**
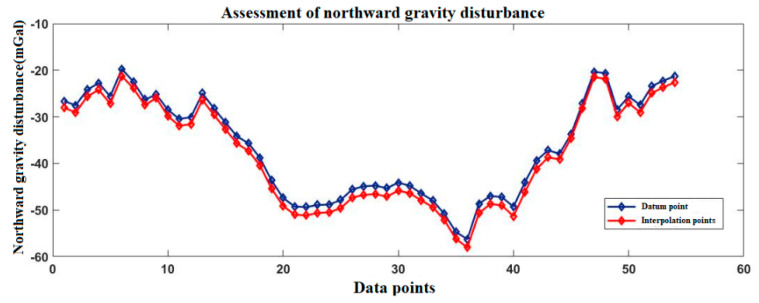
Accuracy evaluation of northward gravity disturbance in the Yanliang area.

**Figure 4:**
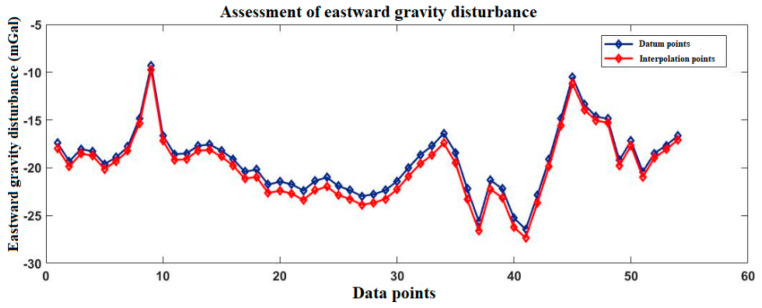
Accuracy evaluation of eastward gravity disturbance in the Yanliang area.

Figure 3 and Figure 4 show the gravity disturbance value at the selected interpolation point and the gravity disturbance value at the corresponding datum point; the gravity disturbance data evaluation at the interpolation point is shown in Table 3.

**Figure 5:**
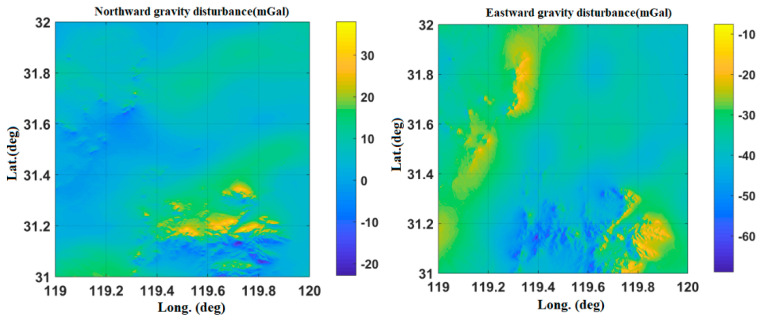
Variation of gravity disturbance in the Changzhou area.

Figure 5 shows the Changzhou gravity disturbance database after interpolation. The highest value of gravity disturbance is about 38 mGal, and the lowest value is about −64 mGal.

**Figure 6:**
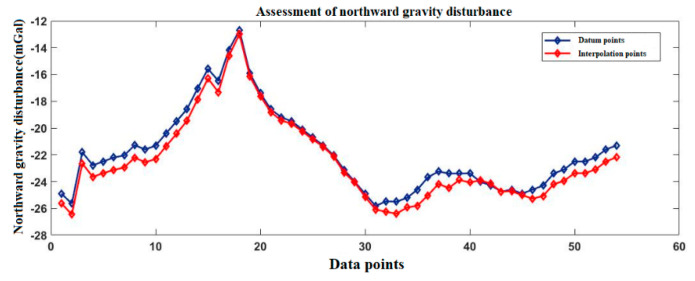
Accuracy evaluation of northward gravity disturbance in the Changzhou area.

**Figure 7:**
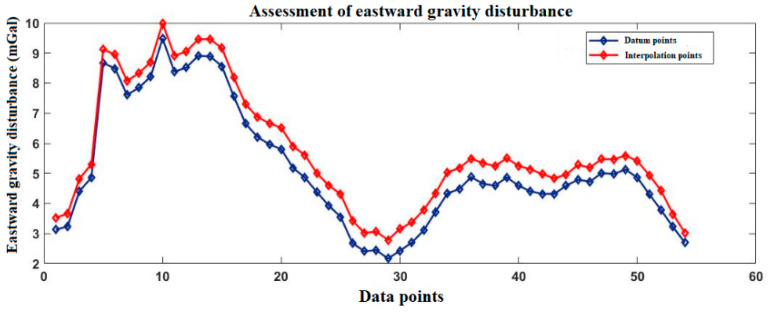
Accuracy evaluation of eastward gravity disturbance in the Changzhou area.

Figure 6 and Figure 7 show the gravity disturbance value at the selected interpolation point and the gravity disturbance value at the corresponding datum point; the gravity disturbance data evaluation at the interpolation point is shown in Table 4.

**Figure 8:**
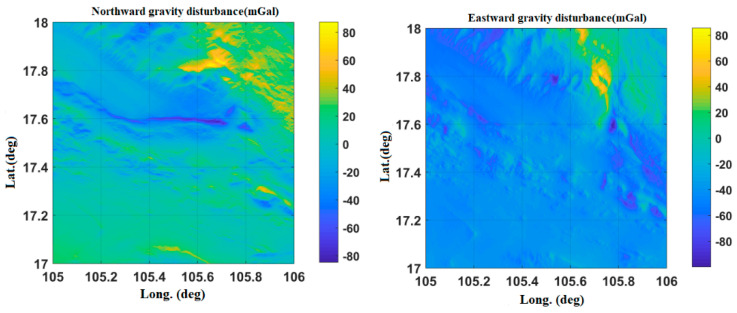
Variation of gravity disturbance in the Hainan area.

Figure 8 shows the Hainan gravity disturbance database after interpolation. The highest value of gravity disturbance is about 82 mGal, and the lowest value is about −94 mGal.

**Figure 9:**
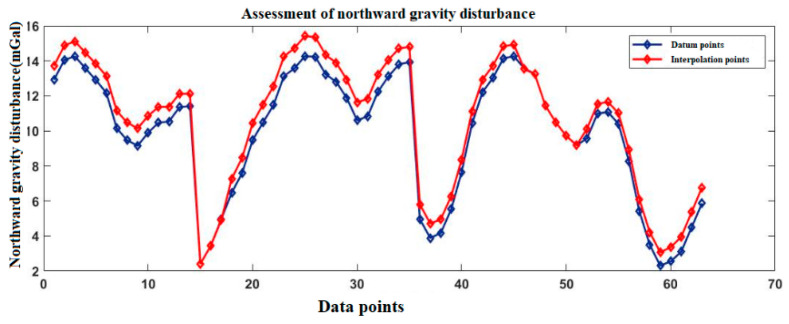
Accuracy evaluation of northward gravity disturbance in the Hainan area.

**Figure 10:**
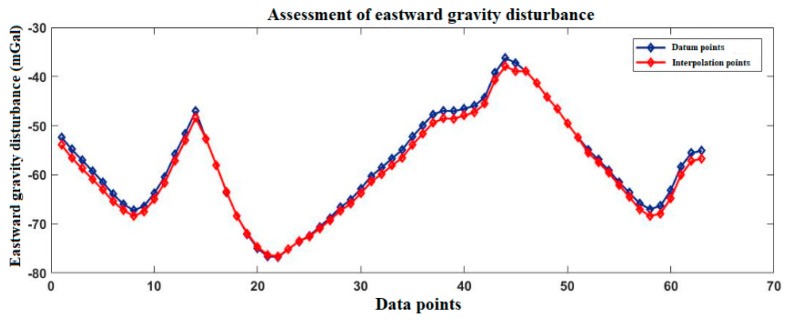
Accuracy evaluation of eastward gravity disturbance in the Hainan area.

Figure 9 and Figure 10 show the gravity disturbance value at the selected interpolation point and the gravity disturbance value at the corresponding datum point; the gravity disturbance data evaluation at the interpolation point is shown in Table 5.

According to Table 3, Table 4 and Table 5, the average error of gravity disturbance in the north and east direction of Yanliang gravity database is 0.76 mGal and −0.58 mGal, respectively, the number of interpolation points with absolute error in the range of −0.8 mGal~0.8 mGal accounts for 96.2%. Changzhou is −0.58 mGal and 0.32 mGal, the number of interpolation points with absolute error in the range of −0.8 mGal~0.8 mGal accounts for 96.4%. Hainan is −0.39 mGal and 0.72 mGal, the number of interpolation points with absolute error in the range of −0.8 mGal~0.8 mGal accounts for 95.3%. After interpolation, the database meets the accuracy requirements.

To sum up, the average gravity database is mainly from the network smoothing method and the fusion of locally measured data. After the data processing of this layer, the gravity database architecture is still in the form of a grid, but its resolution increases from 7.2 “to 2.4”.

## 3. B-TV-MM

The gravity disturbance error model in this section adopts the Block Time-Varying first-order Markov gravity disturbance error Model (B-TV-MM) proposed in reference [9], which is improved from the traditional first-order Markov model. The first-order Markov model is difficult to accurately describe the gravity disturbance changes of the earth or the local area of the earth, resulting in the model accuracy not meeting the requirements. Although the accuracy of the second-order Markov model is improved, the computation is too large to meet the real-time requirements. First of all, B-TV-MM is based on the high-precision gravity database to block the earth or the local area of the earth. Then, according to the gravity disturbance error characteristics of each region block, the Block first-order Markov gravity disturbance error Model (B-MM) is constructed.

The autocorrelation function and the power spectral densities function of the first-order Markov model are shown as follows:

(2) $$\left\{ \begin{array}{l} C_{x}(\tau )=\sigma^{2}e^{-\beta \left|\tau \right|}\\ \Phi_{x}(\omega )=\frac{2\sigma^{2}\beta }{\omega^{2}+\beta^{2}} \end{array}\right.$$

where, *σ* is the mean square variance of the state variable, and the *β* is the reciprocal of the time ($\beta =1/\tau_{g}$).

In order to apply it to the Kalman filter, it is written as a state equation:

(3) $$\delta \dot{g}(t)=-\beta \delta g(t)+\sqrt{2\beta \sigma^{2}}w(t)$$

The discretization of Formula (2) leads to the filtering discretization equation of the first-order Markov model:

(4) $$\delta g_{k+1}=(1-\beta \Delta t)\delta g_{k}+\sqrt{2\beta \sigma^{2}}\Delta tw_{k}$$

Equation (4) is the most basic B-MM in B-TV-MM. However, a single first-order Markov model is difficult to accurately reflect the gravity field changes in the whole region. To solve this problem, B-TV-MM selects the corresponding B-MM according to the position information of the carrier in the flight process, and realizes the Block to Block transformation in the flight process to achieve “Time-Varying (TV)”. Using multiple B-MM to fit the whole region, the accurate real-time estimation of the gravity disturbance error of the whole region can be achieved.

## 4. Gravity Disturbance Compensation Device

### 4.1. Design Principles

The main design principles of POS_GDCT (POS Gravity Disturbance Compensation Terminal) are listed below. First, the device applies to a wide range of INS, and its designers do not need to worry about the system error caused by gravity disturbance error. Second, the inherent accuracy of the original INS must avoid reduction in the case of POS_GDCT failure.

POS_GDCT receives the position, altitude, velocity, attitude and other information from the INS, and provides the position, altitude, velocity and attitude compensation for them in real-time based on the real-time compensation method of gravity disturbance [13]. Therefore, POS_GDCT only corrects the output of the inertial measurement/navigation system in real-time and does not feed back to or update the inertial instrument parameters in the inertial measurement/navigation system.

### 4.2. Design Scheme

**Figure 11:**
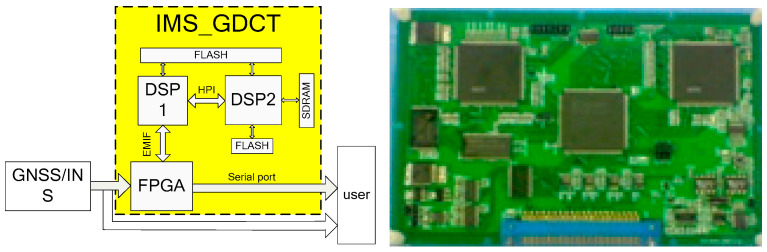
Design block diagram of the device.

Based on the design principles, the gravity disturbance compensation device is designed as an independent working module, which can work with various inertial measurement/navigation systems through a specially coded connector. The overall schematic diagram and physical drawing of the device are shown in Figure 11.

Considering the requirements of real-time and reliability, the device adopts the hardware structure of FPGA (Field Programmable Gate Array) + Dual DSP (Digital Signal Processing). According to the function, it can be divided into two parts: the data input and output module, and the data-processing module. The data input and output module takes FPGA as the core, which is responsible for collecting all information from the INS and interacting with user data. As for the data-processing module, it takes DSP as the core and applies a distributed Dual DSP parallel structure. DSP1 is the main processor, which can read the locked information from the INS, access to the internal high-precision gravity map data, perform the mathematical operations related to the calculation of inertial measurement/navigation gravity correction, and manage, coordinate and schedule the work process of the whole distributed processing unit. DSP2 is a slave processor, which performs data unpacking, data integrity detection, gravity disturbance compensation method calculation and other tasks. The data between DSP1 and DSP2 is transmitted at a high speed and parallels through the Host Port Interface (HPI). During the process of data transmission, DSP is not required to participate in and DSP solution is not interrupted.

In addition, the decoding circuit of the whole system, the coordination circuit between DSP and the interrupt application control circuit are all implemented in FPGA, which reduces the size of the circuit board and increases the flexibility of the circuit design.

### 4.3. Hardware Design

(1) Data input and output module

The data input and output module takes FPGA as the core processing unit. The FPGA is developed with XC3S400-4I of Xilinx Company and VHDL (Very-High-Speed Integrated Circuit Hardware Description Language).

FPGA expands a serial port to receive the data from GNSS/INS, and stores the target data in the corresponding data buffer (FPGA_GNSS/INS_BUF). After receiving a frame of data, it applies to DSP1 for interruption and waits for DSP1 to read. Data output is to output the compensated position, speed and attitude information to the user or upper computer through the serial port. The serial port is also expanded by FPGA. DSP1 continuously writes the data, which need to be delivered to the FPGA serial port data buffer (FPGA_USER_BUF), and then FPGA sends the data to the user or upper computer in turn.

(2) Data-processing module

The data-processing module adopts the cooperative working mode of dual DSPs. The DSP adopts the high-performance floating-point chip TMS320C6713B-A200 of TI Company, the highest main frequency of which is 200 MHz. When working at the highest main frequency, the processing capacity is 1600MIPS/1200MFLOPS. Furthermore, 256KB RAM (Random Access Memory) is integrated into the DSP chip, of which 64 KB is used as a cache, and the remaining 192 KB is enough for user programs. In order to facilitate data interaction between DSP1 and DSP2, DSP2 expands a piece of SDRAM (Synchronous Dynamic Random Access Memory). DSP1 and DSP2 share a 2 M × 8 bit flash chip for storing user programs; in addition, DSP2 expands a 16 GB × 16 bit FLASH chip for storing the gravity value of the earth area.

**Figure 12:**
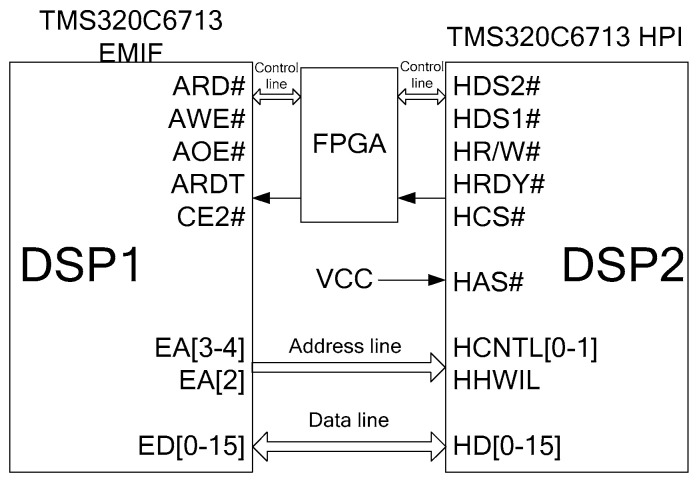
External memory interface (EMI) and host port interface (HPI) interface diagram of Dual Digital Signal Processing (DSP).

The parallel HPI is used for data interaction between the two DSPs. The main port HPI of C6713 is a 16 bit wide parallel interface, and the external host can directly access to the storage space of DSP (including mapped peripheral devices) through HPI, so the data transmission process does not interrupt the operation of DSP. In this scheme, DSP1 is the host and DSP2 is the slave. DSP1 is connected with DSP2 (HPI) through the external memory interface (EMIF) (as shown in Figure 12) to realize the data exchange between two DSPs.

### 4.4. Algorithm Flow

**Figure 13:**
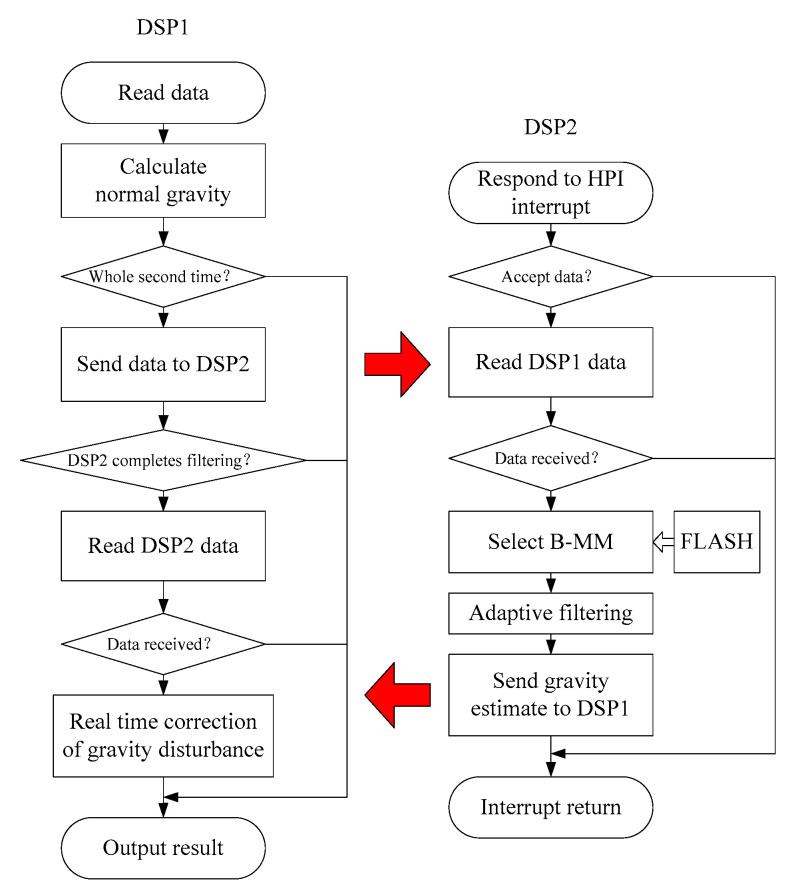
Software flow chart.

The flow chart of the device software is shown in Figure 13. The left side is the algorithm flow of DSP1, and the right part comes to the algorithm flow of DSP2.

### 4.5. Structural Design

**Figure 14:**
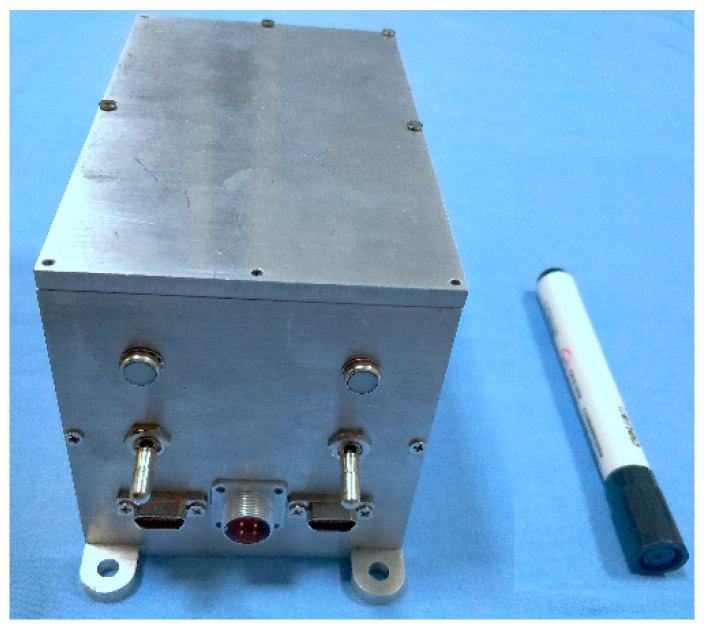
Gravity disturbance compensation device.

The structure of the device is shown in Figure 14, with the overall dimension of 100 mm × 90 mm × 90 mm. The shell material is aluminum 7050 with high strength and strong corrosion resistance, and the shape is hexahedron structure. Four protruding holes on the bottom of the device are used for fixing on the relevant platform to ensure the stability of the device. There are three layers of structural parts on the front panel of the device. The first layer is the indicator light, which is composed of the left side equipping with the power indicator light and the right part assembled with the working status indicator light of the device. The second layer is the switch with the left side being its main power switch, and the right side of this layer is the algorithm switch, which is mainly used to switch different algorithms in the process of the prototype test. This switch is not available in the formal product. As for the third layer, its left side is the data transmission interface, its middle part is a power interface, and the right one is the program download interface.

## 5. Experiment

### 5.1. Experiment Equipment

**Figure 15:**
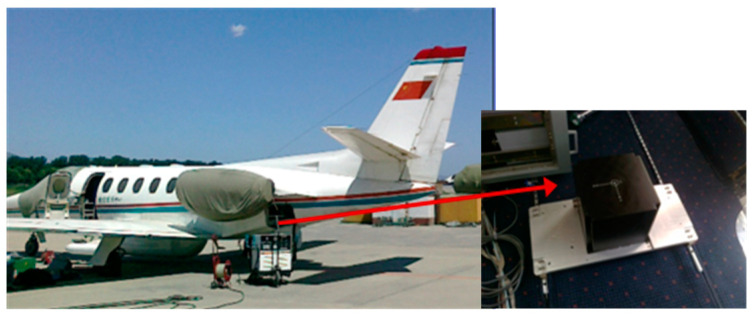
Yun7 airplane and high accuracy POS.

**Figure 16:**
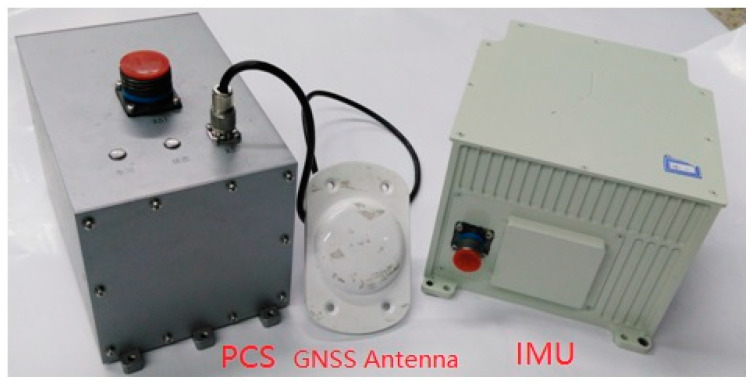
High-precision TX-R610 POS products.

In order to verify the application effect of the POS_GDCT in the aviation field, flight experiments are carried out. Yun-7 airplane was selected as the flight platform (Figure 15). A high-precision Fiber Optic POS (TX-R610) (Figure 16) developed by BeiHang University is selected as the inertial measurement system. Its performance index is shown in Table 6.

### 5.2. The Plan of Flight Experiment

**Figure 17:**
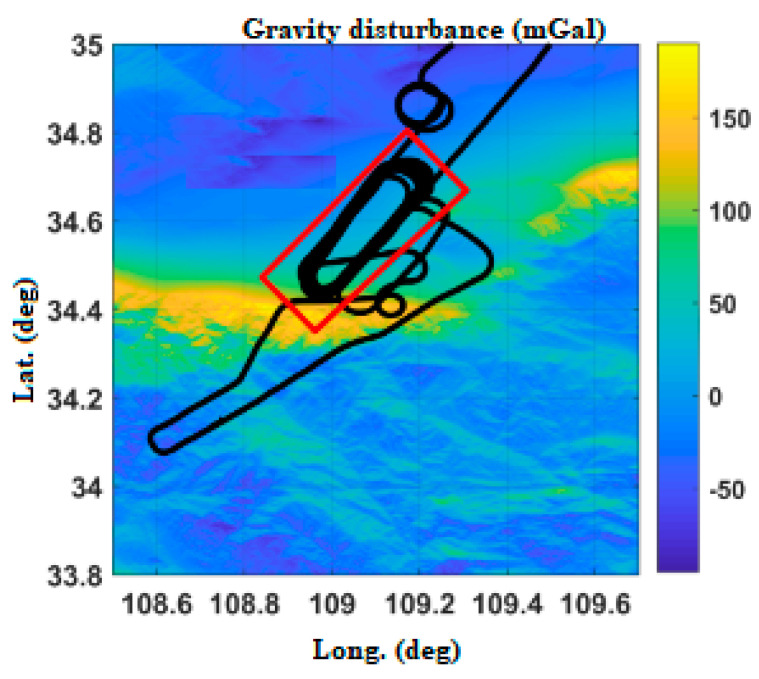
Flight path of Yanliang.

**Figure 18:**
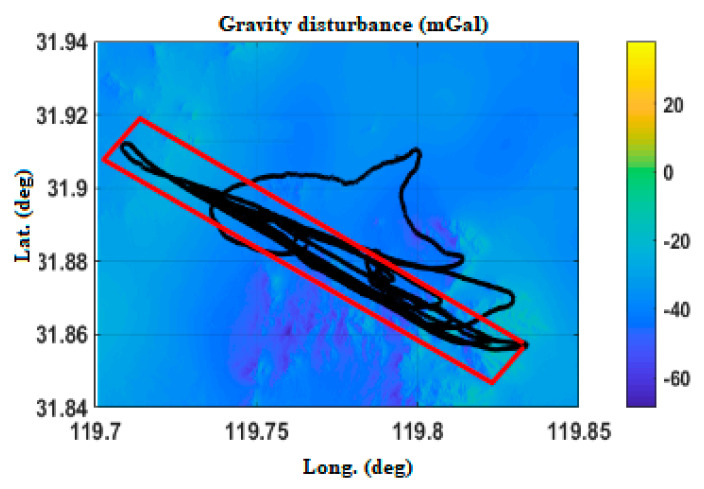
Flight path in Changzhou.

**Figure 19:**
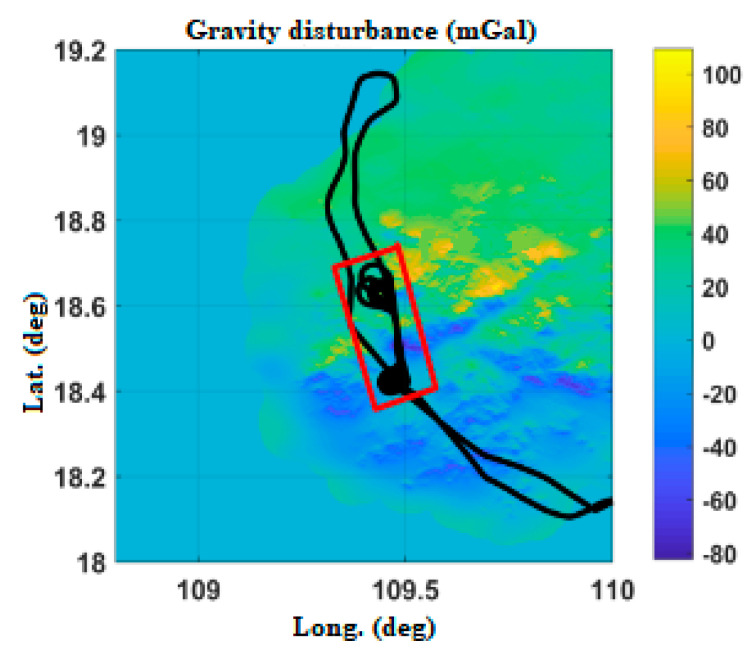
Flight path in Hainan.

POS_GDCT equipment experiment is not a special flight test; it is based on the carrying experiment of other flight missions. The Yanliang region (Figure 17), Changzhou region (Figure 18) and Hainan region (Figure 19) in China are selected, respectively corresponding to a mountainous region, plain and nearshore zone.

### 5.3. Data Analysis and Results

In order to verify the effect of the gravity compensation method, the data of the operation area of the flight path are mainly selected (as shown in the red rectangle of Figure 17, Figure 18 and Figure 19). With POS post-processing measurement results as a test benchmark, the errors of POS real-time attitude measurement under normal gravity model and real-time gravity disturbance real-time compensation device are compared.

Three groups of flight data are chosen for each experimental area to compare the POS real-time attitude measurement accuracy under normal gravity model and gravity disturbance compensation, and the results are shown in Table 7. With the real-time compensation of gravity disturbance, the accuracy of pitch angle, roll angle and heading angle in the Yanliang area increased by 27.6%, 26.2% and 14.4% respectively. In the Changzhou area, the accuracy of pitching angle, roll angle and heading angle increased by 17.6%, 16.2% and 8.4% respectively. In Hainan, the accuracy of pitch angle, roll angle and heading angle increased by 24.2%, 27.1% and 6.2% respectively.

## 6. Conclusions

Gravity disturbance compensation technology remains a research hotspot in the field of pure inertial navigation technology, such as underwater navigation. However, with the application of inertial technology into the field of precise measurements, such as POS, it becomes the core equipment of the high-resolution aerial earth observation system. POS is a typical SINS/GNSS combined measurement system whose attitude accuracy mainly depends on SINS. The vertical component of the gravity disturbance vector is introduced into the POS horizontal channel by vertical deflection, which leads to the POS attitude measurement error.

In this paper, the gravity database architecture for high-precision inertial measurement technology is proposed, and a high-precision gravity database is generated based on different data sources. The accuracy of the gravity database is better than 1 mGal, which meets the requirements of gravity disturbance compensation technology. Moreover, in this paper, based on the real-time compensation method of B-TV-MM gravity disturbance [9], a set of special gravity disturbance compensation devices, POS_GDCT, is developed, and the relevant tests are carried out. The test results show that the POS_GDCT device can effectively improve the attitude measurement accuracy of the inertial measurement/navigation system.

## Figures and Tables

**Table 1:** Attitude measurement error caused by gravity disturbance error.

Gravity Disturbance Error (mGal)	Attitude Error (°)	Gravity Disturbance Error (mGal)	Attitude Error (°)
1	0.00011	20	0.0023
5	0.00059	25	0.0029
10	0.0012	30	0.0035
15	0.0018	35	0.0041

**Table 2:** Applanix Position and Orientation System (POS)/AV 610 Index [29] *.

POS/AV610	C/A GPS	RTK	Post-Processing
Roll and Pitch (°)	0.005	0.005	0.0025

* More details please contact authors.

**Table 3:** Accuracy evaluation of gravity disturbance data in the Yanliang area.

Gravity Disturbance Data Source		Mean Value (mGal)	Variance	Proportion of Effective Forecast Data
Northward gravity disturbance	Datum points	−45.33	16.6382	96.2%
	Interpolation points	−44.87	15.9361	
Eastward gravity disturbance	Datum points	−24.83	9.2472	
	Interpolation points	−25.35	10.7153	

**Table 4:** Accuracy evaluation of gravity disturbance data in the Changzhou area.

Gravity Disturbance Data Source		Mean Value (mGal)	Variance	Proportion of Effective Forecast Data
Northward gravity disturbance	Datum points	−22.64	9.4371	96.4%
	Interpolation points	−23.13	10.4318	
Eastward gravity disturbance	Datum points	5.47	5.2719	
	Interpolation points	5.79	5.8332	

**Table 5:** Accuracy evaluation of gravity disturbance data in the Hainan area.

Gravity Disturbance Data Source		Mean Value (mGal)	Variance	Proportion of Effective Forecast Data
Northward gravity disturbance	Datum points	10.43	4.6281	95.3%
	Interpolation points	10.76	4.9416	
Eastward gravity disturbance	Datum points	−55.63	21.8427	
	Interpolation points	−56.14	22.2875	

**Table 6:** TX-R610 POS product performance list.

Sensors	Parameters	Accuracy (Real Time)	Accuracy (Post Processing)
POS Accuracy	Rate	200 Hz	200 Hz
	Roll and Pitch	0.005° (RMS)	0.0025° (RMS)
	Heading	0.03° (RMS)	0.005° (RMS)

**Table 7:** Attitude error statistics.

POS Index		Yanliang Area		Changzhou Area		Hainan Area	
		NG.	GC.	NG.	GC.	NG.	GC.
1	HA. (°)	0.04d	0.0379	0.0353	0.0281	0.0335	0.0281
	PA. (°)	0.0052	0.0037	0.0062	0.0042	0.0055	0.0042
	RA. (°)	0.0056	0.0039	0.0054	0.0041	0.0043	0.0032
2	HA. (°)	0.0461	0.0359	0.0291	0.0264	0.0278	0.0239
	PA. (°)	0.0058	0.0039	0.0058	0.0042	0.0049	0.0038
	RA. (°)	0.0058	0.0042	0.0043	0.0031	0.0039	0.0031
3	HA. (°)	0.0367	0.0302	0.0264	0.0221	0.0341	0.0301
	PA. (°)	0.0057	0.0039	0.0049	0.0038	0.0056	0.0047
	RA. (°)	0.0053	0.0035	0.0045	0.0036	0.0052	0.0041

HA. = Heading angle, PA. = Pitch angle, RA. = Rolling Angle, NG. = Normal Gravity, GC. = Gravity Compensation.
